# Influence of Cardiometabolic Risk Factors on Platelet Function

**DOI:** 10.3390/ijms21020623

**Published:** 2020-01-17

**Authors:** Cristina Barale, Isabella Russo

**Affiliations:** Department of Clinical and Biological Sciences, Turin University, 10043 Orbassano (Turin), Italy; cristina.barale@unito.it

**Keywords:** adipose tissue, adipokines, hemostasis, insulin resistance, metabolic syndrome, nitric oxide, oxidative stress, platelets, thrombosis

## Abstract

Platelets are key players in the thrombotic processes. The alterations of platelet function due to the occurrence of metabolic disorders contribute to an increased trend to thrombus formation and arterial occlusion, thus playing a major role in the increased risk of atherothrombotic events in patients with cardiometabolic risk factors. Several lines of evidence strongly correlate metabolic disorders such as obesity, a classical condition of insulin resistance, dyslipidemia, and impaired glucose homeostasis with cardiovascular diseases. The presence of these clinical features together with hypertension and disturbed microhemorrheology are responsible for the prothrombotic tendency due, at least partially, to platelet hyperaggregability and hyperactivation. A number of clinical platelet markers are elevated in obese and type 2 diabetes (T2DM) patients, including the mean platelet volume, circulating levels of platelet microparticles, oxidation products, platelet-derived soluble P-selectin and CD40L, thus contributing to an intersection between obesity, inflammation, and thrombosis. In subjects with insulin resistance and T2DM some defects depend on a reduced sensitivity to mediators—such as nitric oxide and prostacyclin—playing a physiological role in the control of platelet aggregability. Furthermore, other alterations occur only in relation to hyperglycemia. In this review, the main cardiometabolic risk factors, all components of metabolic syndrome involved in the prothrombotic tendency, will be taken into account considering some of the mechanisms involved in the alterations of platelet function resulting in platelet hyperactivation.

## 1. Introduction

**Figure 1:**
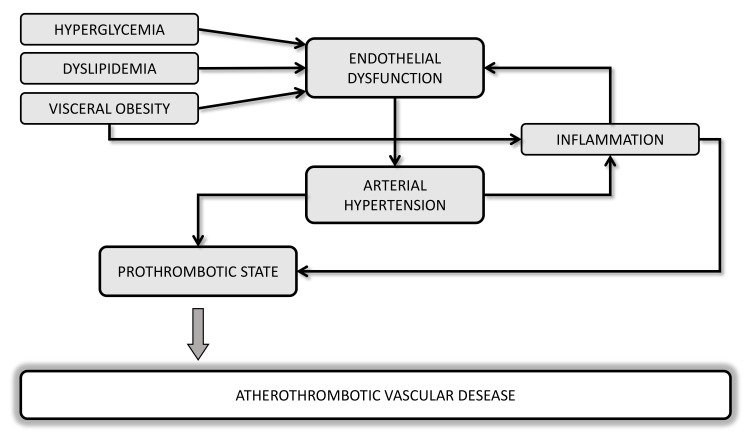
Potential mechanisms linking cardiometabolic disorders and atherothrombotic vascular diseases.

Several lines of evidence suggest a strong correlation between metabolic disorders and hemodynamic such as obesity, dyslipidemia, diabetes, hypertension, and cardiovascular (CV) diseases (CVD), with endothelial dysfunction as the initial step toward atherothrombosis (Figure 1). Oxidative stress and a chronic low-grade of inflammation may be considered a “common soil” able to create a feed-forward cycle that can deeply influence the development of a prothrombotic tendency of these metabolic abnormalities.

One of the first epidemiological studies showing the causal relationship between obesity and CVD was the Framingham Heart Study [1,2] and other studies have then confirmed that the waist-to-hip ratio (WHR)—a reliable index of central obesity—was the strongest anthropometric predictor of myocardial infarction [3] and stroke [4,5].

Not only the excess of adipose tissue, but also body fat distribution and the impaired adipose tissue function, rather than total fat mass, better predict CV risk [6]. Actually, atherothrombotic events leading to an elevated risk of CV morbidity and mortality are closely associated to central obesity, which confers a higher degree of CV risk than peripheral adiposity [7,8]. In fact, abdominal adiposity may have a causal, unfavorable effect on plasma triglycerides (TGs) and potentially other cardiometabolic risk factors due to a greater ability to release cytokines and free fatty acids involved in the pathogenesis of both atherothrombosis and insulin resistance [9,10].

The increasing prevalence of obesity, especially in Western Countries, has also contributed to significant increases in the prevalence of other important CV risk factors, including dyslipidemia, insulin resistance, and type 2 diabetes mellitus (T2DM). The presence of a clustering of three or more risk factors in the same individual, including abdominal obesity, atherogenic dyslipidemia, high systolic and diastolic blood pressures, and impaired glucose tolerance has been defined by World Health Organization (WHO) as metabolic syndrome (MS) [11], though there is some minor variation in the definition by other health care organizations. Importantly, each of these cardiometabolic disorders contributes to alter hemostatic balance leading to a prothrombotic phenotype [12]. This review will focus on the role of obesity on prothrombotic tendency in patients affected by MS, being adipocytes able to produce and/or release hormones which deeply influence hemostatic balance, platelet function, pro-inflammatory state and oxidative stress.

## 2. Definition of Metabolic Syndrome

MS is a multiplex risk factor for atherosclerotic CV disease, with a prevalence of 34% in the general population [13]. However, due to the lacking of a unifying definition, MS can be present in several forms according to the combination of the different components and the exact evaluation of prevalence of MS changes both in United States and in Europe. It has been estimated that at least one quarter of America population is affected by MS and about 84% of them present abdominal obesity on the basis of the criteria indicated by National Cholesterol Education Program (NCEP) Adult Treatment Panel (ATP) III [14,15,16,17,18].

According to the NCEP’s ATP III criteria [19], MS is recognized as a condition related to CVD occurring if the patient has three or more of the following: (1) central obesity characterized by waist circumference >102 cm in men and >88 cm in women; (2) fasting blood TGs ≥150 mg/dL and high-density lipoprotein cholesterol (HDL) ≤40 mg/dL in men or ≤50 mg/dL in women; (3) fasting glucose ≥100 mg/dL; and (4) systolic blood pressure ≥130 mmHg and/or diastolic blood pressure ≥85 mmHg. Indeed, elevated high-sensitivity C-reactive protein, increased prothrombotic factors, endothelial dysfunction, microalbuminuria, elevated inflammatory cytokines, decreased adiponectin plasma levels, and alterations in pituitary-adrenal axis could be involved in MS. However, the inclusion of these abnormalities in the classification of MS needs to be confirmed and this continuous score would be more sensitive to small and large changes that do not modify the most recent Joint Interim Statement of the International Diabetes Federation (IDF) Task Force on Epidemiology and Prevention criteria [20].

Despite other definitions also have been proposed [21], all are associated with the presence of central obesity, thus underlining the crucial role of the abdominal adiposity, together with insulin resistance, as causative factor in the pathogenesis of MS. Actually, the condition of insulin resistance represents a significant link among components of MS even if a subject with MS not necessarily is insulin resistant [22]. It is well established that MS is a constellation of cardiometabolic determinants associated with increase not only of CVD but also a three-fold increase in the risk of T2DM [23,24,25] with significant adverse effects on health-related quality of life [26].

## 3. Platelets in Hemostasis and Thrombosis

Platelets are key players in primary hemostasis and thrombus formation. Platelet activation become when platelets come in contact with exposed collagen in the areas of vascular damage and the subsequent morphological and physiological changes help in stable platelet plug formation thus contributing to primary hemostasis. Platelet activation process is mediated by surface exposure of receptors (glycoproteins, GPs) and lipid rafts, which modulate signaling and intracellular trafficking. These include GPIb/V/IX complex, which interacts with von Willebrand factor (vWF), integrin α IIbβ3 (GPIIb/IIIa), which binds vWF and fibrinogen, and GPVI which binds collagen thus ensuring a stable anchorage with subendothelial matrix [27]. Binding of ligands to the GP receptors changes platelet shape as well as triggers the release of platelet granule contents, which lead to the formation of platelet plug.

**Figure 2:**
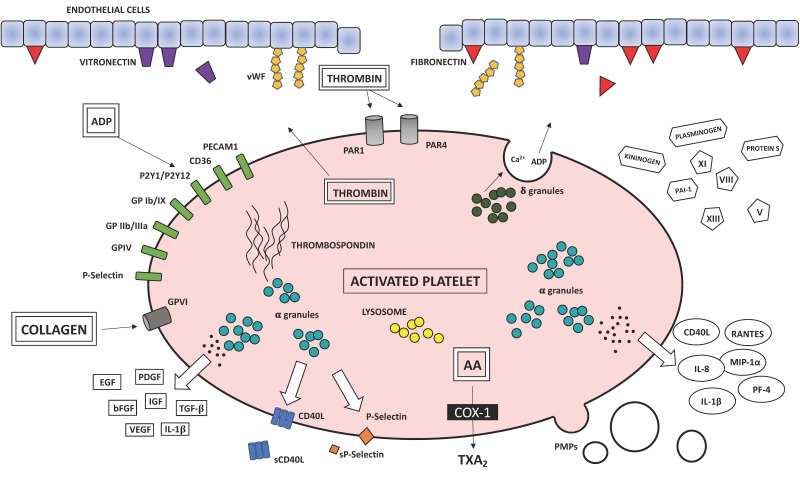
Biochemical factors involved in the coagulation cascade and the atherosclerotic process released following platelet activation. AA, arachidonic acid; COX, cyclooxygenase; TXA_2_, thromboxane A_2_; PDGF, platelet-derived growth factor; TGF-β, transforming growth factor β; EGF, endothelial growth factor; bFGF, fibroblast growth factor; VEGF, vascular endothelial growth factor; IGF, insulin-like growth factor; IL-1β, interleukin-1β; PAI-1, plasminogen activator inhibitor 1; vWF, von Willebrand factor; GP, glycoprotein; PECAM, platelet endothelial cell adhesion molecule; sCD40L, soluble CD40 ligand; sP-selectin, soluble P-selectin; RANTES, regulated on activation, normal T-cell expressed and secreted; MIP-1α, macrophage inflammation protein- 1α; IL-8, interleukin-8; PF4, platelet factor 4; PMPs, platelet-derived microparticles.

However, hemostasis or blood coagulation are not the only function of platelets, which are also involved in pathological processes such as chronic inflammation and atherothrombosis. In fact, platelets store cytokines and growth factors in their alpha-, dense granules and lysosomes [28] and the subcellular machinery of the novo protein synthesis involved in the coagulation cascade and inflammatory pathways including interleukin (IL)-1β, plasminogen activator inhibitor-1 (PAI-1) and tissue factor (TF; Figure 2).

The atherothrombotic process underlies acute coronary and cerebrovascular events where the activation of inflammatory mechanisms is strictly dependent on interaction among different cell types, such as platelets, leukocytes, and cells of the vascular wall. As extensively reviewed [29,30,31], once adhered to the damaged vessel wall platelets participate in multiple mechanisms promoting thromboinflammation by releasing storage granules and aggregating to form thrombi. As mentioned, platelet adhesion is influenced by adhesion molecules present in the subendothelial matrix components, such as E-selectin [32], vWF [33], collagen, fibronectin, and by the level of shear stress in the circulation [34]. In this phase, platelets are subjected to a number of physiological and cytoskeletal changes, with release of soluble cytokines, chemokines, growth factors, and the rapid translocation of P-selectin from alpha-granule to plasma membrane. When intracellular Ca^++^ concentration exceeds a specific threshold, platelets shift from the resting discoid shape to the activated state with the formation of filopodia and lamellipodia. The recruitment of other platelets, their activation, and aggregation are followed by the formation of three-dimensional aggregates for a number of molecular interaction triggered by thrombin and generation of endogenous factors such as thromboxane (TX)A_2_ and release of content from storage granules including adenosine 5-diphosphate (ADP), and platelet activating factor (PAF). Stabilization of platelet–platelet interactions is further mediated by the receptor of fibrinogen GPIIb/IIIa. In the primary hemostasis a pivotal role in plug formation is exerted by platelet aggregation with aggregates anchored at site of injury but this clot remains unstable. Clot stabilization characterizes the secondary hemostasis with consolidation of platelet mass through the assembly of coagulation complexes with conversion of soluble fibrinogen into insoluble fibrin by thrombin and platelet retraction. In some pathological settings, a number of factors can impair the normal hemostasis and aberrant thrombus formation has severe pathological consequences, leading to fatal thromboembolism and tissue ischemia of vital organs, ultimately resulting in acute CVD complications, including myocardial infarction, stroke and critical limb ischemia.

In the presence of intact vascular endothelium, the release of prostacyclin (PGI_2_) and nitric oxide (NO), two major antiaggregants, regulates the balance between pro- and antiaggregants and prevents the formation of thrombus inside the blood vessel [35]. However, in subjects at risk of arterial thrombosis, this key protective pathway is overcome, resulting in uncontrolled platelet activity.

## 4. Platelet Function Assays

For the measurement of platelet function there is no gold-standard method showing the real state of “hyper” or “hypo” reactivity that can be used as reliable marker of high risk in disease settings. However, based on the platelet ability to interact with each other as well as with other cells and for peculiar surface expressions it is possible to measure platelet function and activation by using a number of tools each measuring different aspects of platelet response. Laboratory tests, including light transmission platelet aggregation, lumiaggregometry, impedance aggregometry on whole blood, flow cytometry or enzyme-linked immunoassays (ELISA), are traditionally utilized for the identification of patients with impaired platelet function.

Light transmission aggregometry (LTA), for a long time considered the gold-standard method, is the most widely employed test in clinical hematology to measure the increase in light transmission through a platelet suspension when platelets were stimulated by a specific agonist such as ADP, arachidonic acid (AA), collagen, and epinephrine. LTA allows us to evaluate the tendency of platelets to aggregate and to identify abnormalities such as hyperaggregation [36]. This assay has some major drawbacks: (i) it is relatively non physiological because during the test platelets are stirred under low shear conditions and only form aggregates after the addition of agonists, without mimicking platelet adhesion, activation and aggregation upon vessel wall damage, (ii) the result obtained may be affected by preanalytical and procedural conditions, (iii) not suitable for platelet-rich platelet numbers below 50 × 10^6^/mL and lipemic blood sample, and (iv) its reproducibility is poor. Specific guidelines for LTA have been published in order to correctly perform the procedure [36,37,38].

Platelet aggregation assessed in whole blood (WB) measures the increase of electrical impedance generated by aggregates upon those fixed to two electrodes. As advantages, WB aggregometry evaluates platelet function under more physiological conditions because of the presence of the other blood components, does not require manipulation of the sample, thus avoiding platelet activation, and all platelet subpopulations are present in WB sample [39].

Flow cytometry assay allows the rapid analysis of physical and antigenic properties of platelets, such as presence of platelet aggregates or leukocyte–platelet aggregates, determination of GP receptor expression (i.e., GPIIb/IIIa, GPIb/IX/V, and GPVI), including conformational changes related to the receptor activation (i.e., GPIIb-IIIa), activation markers (CD62P and CD63), and platelet granule secretion (β-thromboglobulin, thrombospondin-1, vWF, fibrinogen, and P-selectin). In WB samples, the use of a double labeling binding allows the identification of platelets, platelet microparticles, or mixed cell aggregates [40,41,42]. Although flow cytometry has the advantages to require small volume of blood sample, to perform platelet aggregation also in the presence of low platelet counts, and to analyze platelet function and activation in a physiological environment [43], this approach can be affected by preanalytical manipulations and be prone to artifacts [44,45]. ELISA are now the most commonly used assays for the measurement of platelet activation markers TXA_2_ metabolites (serum TXB_2_ and urinary 11-dehydro-TXB_2_) [46] and alpha-granule factors such as β-thromboglobulin, platelet factor (PF)-4, soluble P-selectin (sP-selectin), and soluble CD40 Ligand (sCD40L) [44,45]. The new Point-of-Care devices (i.e., VerifyNow system, Plateletworks, Platelet Function Analyser-100, and Multiplate Electrode Aggregometry) may be useful supplements to the existing well-known platelet function tests and are mainly utilized for monitoring antiplatelet therapies.

## 5. Platelet Alterations in Central Obesity

Obesity is a heterogeneous condition and, when located within the abdominal cavity, becomes an independent determinant for cardiometabolic disease causing or exacerbating other cardiovascular and metabolic risk factors, such as dyslipidemia, hypertension, insulin-resistance, and T2DM [47,48,49]. Apart from metabolic and hemodynamic alterations, central obesity is characterized by a chronic low grade inflammation and systemic oxidative stress that eventually damages the endothelium causing the loss of the endothelium antithrombotic properties. This justifies the assumption of obesity as a pro-thrombotic clinical condition with increased platelet activation and decreased fibrinolysis [50,51,52,53], both contributing to atherogenesis and acute atherothrombotic events via increased vascular deposition of platelets and fibrinous products.

Platelets from obese subjects are known as “angrier” because they show a number of abnormalities, which increase platelet aggregability and activation constituting a relevant risk factor for CVD, especially for the development of atherothrombosis [54]. Recently, studies linking proteomic analysis and aggregation findings have confirmed the presence of alterations in proteins related to platelet signaling [55]. In particular, a higher expression of GPVI, positively correlated with body mass index (BMI), together with higher levels of Src (pTyr418) and tyrosine phosphorylated phospholipase Cγ2, essential for integrin signaling, mechanistically provide possible explanations for platelet hyperreactivity in obesity [55].

Certain adipokines, bioactive peptides secreted by omental adipose tissue, can modulate not only body weight and metabolism but also vascular function [56]. For instance, in the platelet hyperreactivity of obese individuals [57,58,59,60,61], associations with leptin, the satiety hormone produced primarily by the adipose tissue, and adiponectin, an insulin-sensitizing adipokine produced exclusively by adipocytes, have been found. Platelets express the leptin receptor and both leptin and leptin-receptor-deficient mice have been protected from experimental thrombosis [62]. In in vitro experiments with human platelets, leptin alone does not induce platelet aggregation but increases the proaggregating effects of sub-threshold concentrations of ADP and thrombin [63]. A specific pathway in the leptin-induced platelet activation involves Janus kinase 2 (JAK2), phosphatidylinositol 3 kinase (PI3K) and phospholipases Cγ2 and A2, with effects on 3′,5′-cyclic adenosine monophosphate (cAMP) hydrolysis, GPIIb/IIIa expression, and TX synthesis. Furthermore, independently of other risk factors, high plasma levels of leptin are associated with an increased risk of thrombotic events such as acute myocardial infarction and stroke [63].

Differently from the other secretory products of adipocytes, adiponectin exerts anti-inflammatory effects protecting against thrombosis, insulin-resistance, dyslipidemia, and endothelial dysfunction [55]. Adiponectin, the most abundant secretory protein produced by adipocytes, is synthesized and secreted as a trimer and in multimeric complexes cleaved to forms that are active transducer of signaling [64]. In mouse, adiponectin has been shown to increase fatty acid oxidation, perhaps through the activation of AMP kinase (AMPK). Disruption of adiponectin leads to high-fat diet–induced insulin resistance and levels are low in humans with obesity and insulin resistance while adiponectin levels are increased by insulin-sensitizing peroxisome proliferator-activated receptors (PPAR)γ agonists.

Although adiponectin per se does not influence platelet aggregation [65], antithrombotic actions have been attributed to this adipokine. In particular, adiponectin deficient mice show increased platelet response to the proaggregating agents and thrombosis tendency [66], high adiponectin plasma concentrations are associated with a decreased risk of coronary artery diseases and increased bioavailability of NO [67]. Both hyperleptinemia and hypoadiponectinemia in MS are associated with increases in leukocytes and platelet indices with platelet count, platelet distribution width (PDW), mean platelet volume (MPV) values, and platelets/lymphocyte ratio significantly higher in MS patients than in healthy subjects [68].

## 6. In Vivo Markers of Platelet Activation in Obesity

Activated platelets show peculiar features or express certain proteins that are less detectable in resting platelets, thus these factors can be used as markers of platelet activation. Some of these markers are higher in central obesity than in healthy subjects (Table 1).

### 6.1. Mean Platelet Volume 

Among the in vivo markers of platelet activation in obesity, MPV represents a parameter closely related to platelet hyperactivation [69] and it has been found increased in obese subjects [70,71]. An interventional study carried out on female subjects, showed MPV values significantly higher in the group of obese women, in comparison with the non-obese [70]. A positive correlation was found between not only MPV and BMI but also reduced values of MPV and weight loss. Conversely, in another cross-sectional study on male individuals, it was not observed any significant difference in MPV values between groups with abdominal and without it. However, in the same study, MPV displayed a positive correlation with prothrombin activity [72]. Weight loss after bariatric surgery is also accompanied by a decrease in platelet count and significant changes in MPV, especially 6 months after surgery, corresponding to the period when weight loss was at its maximum [73].

### 6.2. Arachidonic Acid Metabolites

TXA_2_ is an unstable platelet-derived proaggregant agent with persistent biosynthesis in several CVD [74]. Precursor of TX synthesis is AA dissociated from membrane phospholipids following the increased Ca^++^ intracellular levels and phospholipases activity [75]. A crucial role in TXA_2_ production is played by the action of the constitutively expressed cyclooxygenase (COX)-1 in platelets and inducible COX-2 in monocytes and other cells in response to inflammatory and mitogenic stimuli. TXA_2_ has a short half-life and is nonenzymatically hydrolyzed and further converted into stable metabolites excreted in the urine 2,3-dinor-TXB_2_ and 11-dehydro-TXB_2._ The urinary excretion of 11-dehydro-TXB_2_, which represents the more reliable time-integrated index of systemic TXA_2_ synthetized for 70% by platelets, has been found increased in women with abdominal obesity and higher in women with android obesity than in those with gynoid obesity [76]. Noteworthy, serum TXB_2_ levels were found lower in insulin sensitive morbidly obese subjects than in the obese subjects and lean subjects, suggesting that reduced platelet activation could play a role in the paradoxical protection of morbidly obese subjects from atherosclerosis, despite the greater levels of leptin and C-reactive protein [77].

Abdominal obesity increases oxidative stress, as demonstrated by the increased levels of lipid peroxidation or protein oxidation products [78]. Indeed, the chronic ‘metabolic inflammation’ [79], the hallmark of obesity causing insulin resistance and T2DM [80], where the metabolic disorders trigger inflammatory signals, contributes to generate reactive oxygen species (ROS), which influence platelet function by different ways. For instance, isoprostanes are a family of products derived from AA metabolism through ROS-dependent mechanisms.

An oxidation product of AA is 8-iso-prostaglandin F_2α_ (PGF_2α_), an abundant isoprostane involved in platelet aggregation by activating TX receptor in the presence of sub-threshold concentrations of other agonists. The influence of this isoprostane on platelets can be prevented by TXA_2_ receptor antagonism but is completely independent of COX-1 activity [29]. A positive linear correlation between urinary excretion of 11-dehydro-TXB_2_ and PGF_2α_ underlines the link of platelet activation with oxidative stress [81].

### 6.3. Soluble P-Selectin

A pivotal role in the development of vascular complication of atherothrombosis is played by cellular adhesion pathways and selectins are one of the four main adhesion molecule families. Platelets are the major source of P-selectin, a cellular adhesion molecule with procoagulant activity [82] and able to activate leukocyte integrins [83]. The circulating levels of soluble form of P-selectin mirror platelet activation. Stored in the alpha-granules of platelets, in a setting of inflammation P-selectin translocates to the plasma membrane where it can interact with ligands [84] leading to leukocyte-platelet aggregates that promote adhesion and infiltration of inflammatory cells [85,86,87,88]. sP-selectin has been associated with adiposity and both clinical and subclinical atherosclerosis [89] and has been shown to predict atherosclerosis independently of BMI and other CVD risk factors. The enhanced plasma concentrations of P-selectin in overweight and obese insulin resistant subjects [61,90] are reduced after weight loss [61].

### 6.4. CD40 Ligand

Activated platelets also release the sCD40L, a trimeric transmembrane protein structurally related to tumor necrosis factor (TNF)-α superfamily. CD40 and its immunomodulating CD40L show dual prothromboting and proinflammatory role further contributing to amplify vascular diseases and atherogenesis [91].

More than 95% of circulating sCD40L derives from platelets, stored in high amounts in cytoplasma in unstimulated platelets, expressed on the platelet surface where it is cleaved to form the soluble trimeric fragment and released within seconds after platelet activation [92]. sCD40L measurement is considered as a platelet-derived marker of cardiovascular risk able to link thrombosis and inflammation [93]. Studies in mice showed that in obesity the genetic or antibody mediated disruption of CD40L signaling ameliorates adipose tissue inflammation and metabolic disorders in insulin resistance [94], thus confirming the role of sCD40L as a platelet-derived marker of the cardiovascular risk able to link thrombosis, inflammation, and altered metabolism [93]. CD40/CD40L interaction is involved in the expression of many proinflammatory and prothrombotic factors, including IL-1, IL-6, IL-8, IL-12, TNF-α, monocyte chemoattractant protein (MCP)-1, and matrix metalloproteinases (MMPs) accelerating the adhesion of monocytes to the vascular endothelium [95,96,97], promoting a ROS-mediated endothelial injury [98,99,100] and the rupture of atheromatous plaques [101]. Recent reports have also indicated that patients with acute cerebral ischemia exhibit increased expression of CD40L on platelets and the CD40/CD40L signaling directly modulates cerebral microvascular thrombosis by the mammalian target of rapamycin (mTOR)/S6K signaling pathway activation [102]. Plasma levels of sCD40 are considered reliable markers of in vivo platelet activation and the increased levels found in obesity are reduced by weight loss [61].

### 6.5. Platelet-Derived Microparticles (PMPs)

Platelet-derived microparticles (PMPs) are small membrane-bound microparticles with a diameter less than 0.1 micron containing bioactive proteins and genetic material (i.e., mRNAs and microRNAs) able to deeply influence phenotypes and functions of recipient cells promoting the development of pathological states [103]. Platelets, activated by various agonists or exposed to high shear stress [104] or increased oxidative stress [57], produce PMPs and elevated levels of circulating PMPs are associated with most of the cardiovascular risk factors including hypertension, obesity, and dyslipidemia [105], appearing indicative of a poor clinical outcome. In obese non-diabetic subjects, elevated circulating levels of PMPs positively correlate with BMI and waist circumference [106]. Weight reduction, by calorie restriction with or without exercise [106] or after gastrectomy, reduces PMP production. Interestingly, another study has recently shown that PMPs from obese subjects were not different in number if compared with non-obese subjects but, as supported by proteomics data, they showed greater heterogeneity in size and distribution with different levels of proteins relevant to thrombosis and tumorigenesis [107].

## 7. Contribution of Insulin Resistance on Platelet Dysfunction

**Figure 3:**
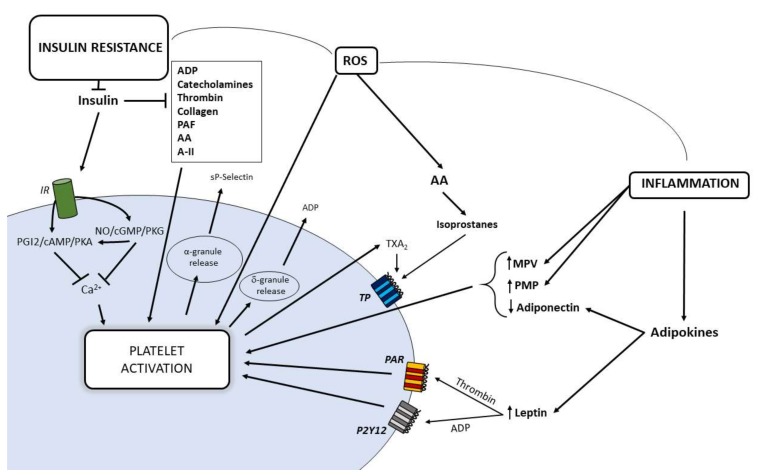
Relationships between insulin resistance, increased oxidative stress and inflammation in promoting platelet hyperactivation in obesity. AA, arachidonic acid; A-II, angiotensin-II; cAMP, 3′,5′-cyclic adenosine monophosphate; cGMP, 3′,5′-cyclic guanosine monophosphate; IR, insulin receptor; MPV, mean volume platelet; NO, nitric oxide; PAF, platelet activating factor; PAR, protease-activated receptor; PGI_2_, prostaglandin I_2_; PKA, cAMP-dependent protein kinase; PKG, cGMP-dependent protein kinase; ROS, reactive oxygen species; PMP, platelet-derived microparticles; TP, thromboxane receptor; TX, thromboxane.

Insulin is a hormone that mediates its action through the insulin receptor (IR) composed of two monomers comprising an extracellular α-subunit and a transmembrane β-subunit [108]. Insulin binding induces IR autophosphorylation at various tyrosine residues, recruitment of IR substrates (IRS), and activation of mitogen-activated protein kinase (MAPK) and PI3K [109]: the activation of these signaling pathways promotes downstream processes involved in blood glucose control [110]. A less than expected response of target organs to insulin leads to a condition of insulin resistance with hyperinsulinemia for a compensatory increased insulin production by pancreatic β-cells. Insulin-resistance is classically referred to metabolic homeostasis characterizing, in most cases, obesity, impaired glucose tolerance and T2DM [111]. Indeed, insulin resistance involves also the vascular effects of the hormone [112,113,114] and it is the common soil of a cluster of metabolic, hemodynamic, thrombotic and inflammatory features deeply involved in atherogenesis and CVD [115]. One of the alterations accounting for the association between insulin resistance and vascular diseases is platelet hyperactivation, also explained by the reduced sensitivity to the physiological and pharmacological antiaggregating agents. Platelet membrane shows functional IR with a density similar to that measured in other target cells of insulin action [116]. In platelets from insulin sensitive subjects, the hormone decreases in vitro platelet aggregation stimulated by common platelet agonists such as ADP, thrombin, catecholamines, PAF, collagen, AA, and angiotensin-II [117,118]. Insulin infusion in euglycemic conditions determines: (i) reduced sensitivity to multiple agonists and deposition to collagen [119]; (ii) impaired primary hemostasis under high shear stress [119]; and (iii) reduced TXA_2_ metabolite synthesis also in T1DM [120]. Through NO increase, insulin induces a rapid increase of the cyclic nucleotides 3′,5′-cyclic guanosine monophosphate (cGMP) and cAMP with inhibitory effects on platelet aggregation [121]. In conditions of insulin resistance such as central obesity, T2DM with obesity and hypertension, the inhibitory effects of insulin on platelets are impaired [53] (Figure 3). Among the mechanisms involved in the altered insulin actions on platelets, a role is played by the effects on platelets of the abnormal adipokine content in plasma profile of patients with MS and T2DM [122]. In particular, the adipokines resistin, leptin, PAI-1, and retinol binding protein 4 (RBP4) induce insulin resistance in megakaryocytes by interfering with IRS-1 expression with a negative impact on insulin signaling in platelets.

Platelets from obese insulin-resistant individuals are characterized by multi-step defects at level of NO/cGMP/protein kinase cGMP-dependent (PKG) and PGI_2_/cAMP/protein-kinase cAMP-dependent (PKA) pathways. In particular, platelets show an impaired NO and PGI_2_ ability to increase, respectively, cGMP and cAMP synthesis and a resistance to cGMP and cAMP themselves to activate their specific kinases PKG and PKA [59,60]. Since the cyclic nucleotides exert their effects on platelets mainly through a reduction of intracellular Ca^++^ [123], these data are suggestive for the presence of alterations in Ca^++^ fluxes handling. Actually, elevated cytosolic Ca^++^ concentrations have been found in insulin-resistance states [124] and this could explain the defective action of cyclic nucleotides on platelet function. Of note, this multistep resistance is not emphasized by the presence of T2DM [125] as well as the presence of T2DM without obesity is not associated with this cluster of platelet abnormalities [125]. However, lifestyle interventions aiming to reduce body weight by diet can modify the prothrombotic tendency in obese insulin resistant individuals. Actually, the altered platelet sensitivity to NO/cGMP/PKG and PGI2/cAMP/PKA pathways in obesity is restored by weight reduction of at least 10% of the initial body weight and this phenomenon is also accompanied by an improvement of insulin resistance and a decrease of markers of inflammation [61] and synthesis of isoprostanes [126]. The central role of the insulin resistance associated with obesity as a pathogenic factor deeply involved in the impairment of the main inhibitory mechanisms of platelet function is confirmed, in the same study, by multiple regression analysis showing the homeostasis model assessment (HOMA) index, a surrogate marker of insulin-resistance, as the parameter more strongly associated with platelet response to the antiaggregating agents. Successful weight loss obtained with drugs, such as the incretin-based therapy, is associated with a significant reduction in TX-dependent platelet activation, possibly mediated, at least in part, by decreased inflammation and lipid peroxidation [127]. In particular, a direct role on platelets by Liraglutide, an analog of the incretin hormone glucagon-like peptide 1 (GLP-1), initially used for the treatment of T2DM and recently introduced as potential weight loss medication, cannot be excluded because Liraglutide has been shown to inhibit platelet activation in animal models [128] and human platelets [129].

## 8. Type 2 Diabetes Mellitus and Alterations of Platelet Function

Platelets from diabetic patients are more prone to form spontaneous microaggregates [130], to adhesion, to aggregation in response to agonists [131], and to be less sensitive to antiaggregants [132]. Biochemical abnormalities associated with these impairments of platelet function can be detected by elevation of intracellular calcium levels and expression of platelet activation markers including PMPs, which in patients with T2DM can be used as potential predictors of CV outcomes [133].

**Figure 4:**
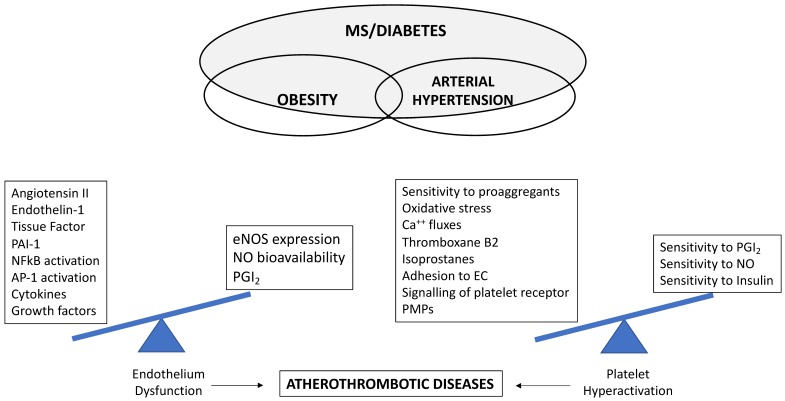
Biochemical imbalance towards factors promoting endothelial dysfunction and platelet hyperactivation involved in the development of atherothrombotic diseases in the presence of multiple cardiometabolic risk factors. eNOS, endothelial nitric oxide synthase; NFκΒ, nuclear factor kappa Β; PAI-1, plasminogen activator inhibitor-1; AP-1 activator protein-1; NO, nitric oxide; PGI_2_, prostaglandin I_2_; EC, endothelial cell; PMPs, platelet-derived microparticles; MS, metabolic syndrome.

Indeed, several mechanisms are involved in the hyperactive platelet phenotype in diabetic patients. Among them, hyperglycemia, oxidative stress, and altered shear stress, interconnected with associated metabolic conditions (obesity, dyslipidemia, and subclinical inflammation) promote atherogenesis and the tendency to a prothrombotic status (Figure 4), which in T2DM represents an important risk factor for a first CV event and for worse outcomes after a CV event.

### 8.1. Hyperglycemia

Although some abnormalities in platelet function in T2DM depend on the presence of the insulin resistance condition, some defects occur only in T2DM in relation to hyperglycemia. Hyperglycemia, the basic characteristic feature of diabetes, and glycemic variability are predictive determinants of platelet activation [134] and postprandial hyperglycemia is an independent risk factor for cardiovascular complications [135]. Although the underlying pathogenic mechanisms are multiple, factors promoting oxidative stress are unanimously considered to contribute significantly to platelet activation. Of particular interest, in T2DM patients a marked oxidative response is induced by the consumption of high-calorie meals, which in these individuals determines an abnormal and sustained elevation of blood glucose and lipid levels, mainly TGs, defined as postprandial dysmetabolism [136].

Since the entry of glucose into platelets does not depend on insulin, intraplatelet glucose concentration mirrors blood glucose levels, and chronic hyperglycemia has been clearly identified as a causal factor leading to platelet hyperreactivity, as indicated by enhanced aggregation, increased fibrinogen binding, and TX production [137]. Hyperglycemic spikes trigger ischemic cardiovascular complications in T2DM [138,139,140] and may elicit arterial thrombosis owing to a transient hyperreactivity of platelets to high shear stress, thus contributing to precipitating arterial thrombotic occlusion at stenotic sites [141]. Furthermore, platelet activation due to high glucose exposure in the absence or in the presence of high shear stress conditions is cause of reduced platelet sensitivity to inhibition by aspirin [142,143,144]. Recently, a reduced acetylation level of the catalytic Ser529 site associated with an incomplete inhibition of COX-1 activity by aspirin has been found in condition of high glucose and diabetes [145], adding another piece of information, which may contribute to explain the residual platelet hyperreactivity observed in diabetes and implying in T2DM the use of effective therapeutic strategies able to prevent hyperglycemia in order to improve also the protective effects of aspirin against the occurrence of CV events.

Platelet hyperreactivity in T2DM is coupled with biochemical evidence of persistently increased TX-dependent platelet activation [137,146] and in the mechanism by which platelets transduce glucose levels into enhanced TX generation a central role is played by the enzyme aldose reductase, the first enzyme of the polyol pathway. The activity of aldose-reductase is significantly enhanced in vascular cells in T2DM and is thought to contribute to vascular complications by increasing oxidative and osmotic stress. Glucose flux through aldose reductase enzyme generates oxidative stress by distinct mechanisms, including nicotinamide adenine dinucleotide phosphate (NADPH) depletion, decrease of glutathione (GSH) levels, and increase of advanced glycation end products (AGEs), thus promoting ROS formation [147]. ROS also play an important role in signaling upon agonist-induced platelet aggregation, inducing changes in intraplatelet Ca^++^, and acting as second messenger in thrombin- or collagen-activated platelets [148]. The increased oxidative stress derived from hyperglycemia and platelet activation potentiates p38α MAPK/cytosolic phospholipase A_2_ signaling, which catalyzes AA release and TXA_2_ production. T2DM with enhanced biosynthesis of TX despite aspirin therapy may have underlying endothelial damage and thromboembolic disease [149]. As mentioned earlier, sCD40 is both marker and mediator of platelet activation and its upregulation is involved in the advanced stage of cerebrovascular disease and increased risk of CV events in T2DM. The increased TX-dependent platelet activation is also associated with enhanced CD40L release [150].

Although mechanisms underlying the pathogenesis of ischemia/reperfusion injury are particularly complex and multifactorial, there is evidence of interactions between platelet function and ischemia/reperfusion injury, especially in diabetic conditions [151].

Diabetic heart is among the most susceptible to ischemia/reperfusion injury and some cardioprotective strategies are compromised in the presence of diabetes because of several mechanisms, including alteration at the mitochondrial level, altered production of ROS, and impairment of antioxidant capacities at various intracellular and extracellular sites [152].

Interestingly, a recent study has shown that the infusion of platelets from healthy subjects in rat isolated hearts exerts cardioprotective effects by reducing infarct size [153] with a mechanism that depends on the platelet capacity to activate cardiac sphingosine-1-phosphate (S1P) receptors and extracellular signal-regulated kinase (ERK)/PI3K/protein kinase C (PKC) pathways. However, platelets from poorly controlled T2DM subjects, as mirrored by high values of glycated hemoglobin (HbA1c), lost their cardioprotective effects, released less S1P, and a positive correlation between infarct size and the amount of ROS produced by diabetic platelets was found [153].

High glucose levels were also found to cause in platelets loss of function and damage to mitochondria, mitochondrial membrane potential dissipation, cytochrome c release, caspase-3 activation, and a subgroup of platelets can undergo apoptosis [154]. Enhanced rate of platelet apoptosis can lead to generation of PMPs that carry thrombotic mediators by providing a new prothrombotic interface for the deposition of fibrin and other blood cells [155].

In addition to up-regulated pro-aggregatory stimuli, platelets from diabetic individuals show reduced sensitivity to the antiaggregating insulin, NO, and PGI_2_ [58]. Since some antiplatelet effects of aspirin are related to increased platelet NO synthesis [144] and preservation of NO from its inactivation [156], an impaired platelet sensitivity to NO signaling may account, at least partially, for less protective aspirin effects against thrombotic events in T2DM.

However, the superoxide-mediated impairment of NO effects on platelet function following hyperglycemia can be corrected by acute aggressive glycemic control [157]. Platelet exposure to high glucose also influences the biophysical state of platelet membrane components and changes in fluidity owing to glycation or acetylation of membrane proteins contribute to the intensified intraplatelet Ca^++^ mobilization [158]. High cytosolic Ca^++^ levels deeply influence the procoagulant state of platelet aggregates inducing externalization of phosphatidylserine and thus accelerating the membrane-dependent reactions of blood coagulation [159].

### 8.2. Oxidative Stress

As mentioned, superoxide radicals have a strong effect for activating platelets and in T2DM oxidative stress is increased for the imbalance between ROS production and antioxidant defenses. High concentrations of ROS influence platelet function by different mechanisms, including decreased NO bioavailability, calcium mobilization abnormalities, over-expression of membrane glycoproteins, and isoprostane formation. A major source of platelet ROS is the enzyme NADPH oxidase (Nox), as demonstrated in platelets from patients affected by chronic granulomatous disease, a rare primary immunodeficiency, that show very low ROS generation and, in the most frequent form, the deficiency of Nox2 subunits. Nox2 is expressed by platelets and its increased activity has been shown to be correlated with platelet activation, isoprostane formation and/or NO inhibition [160,161]. Nox2 activation, platelet recruitment, and isoprostane levels are parallelly increased in diabetic patients and these could be cause of reduced efficacy of aspirin [162].

Oxidation reactions are elevated in patients with T2DM and significantly contribute to form isoprostanes, which are produced from AA through a non-enzymatic process of lipid peroxidation, catalyzed by oxygen-free radicals on cell membranes [163]. Since structurally similar to prostaglandins, once released isoprostanes activate the same receptors. 8-iso-PGF_2α_ influences some aspects of platelet function such as adhesive reactions and activation by low concentrations of other agonists [134]. In poorly controlled diabetes, plasma levels of 8-iso-PGF_2α_ are increased and correlate with impaired glycemic control and enhanced lipid peroxidation, thus providing a biochemical link between impaired glycemic control and persistent platelet activation [164].

### 8.3. Shear Stress

The increased tendency of platelets from diabetics to aggregate is tightly regulated not only by the diabetic milieu but also by complex conditions of flow dynamics. Thrombotic complications are deeply influenced by the effects of hemodynamic environment at the site of vessel injury or plaque rupture blood on endothelial cells constantly exposed to multiple physical forces generated by the movement of blood. In normal conditions, the physiological shear stress-induced endothelial release of NO and PGI_2_ does not allow platelets to adhere to the vessel wall [165,166]. In response to abnormal blood flow endothelial cells can modify their shape, function and gene expression, which, in turn, affect platelets, whose adhesiveness and activation change. High shear rates (>1000 s^−1^) promote platelet aggregation critically modulated by vWF, endogenously present in the subendothelial matrix or absorbed onto injured tissue components exposed to plasma [167], and subjected to conformational changes that determine vWF self-association and vWF fiber formation [168]. Furthermore, vWF activation also requires the formation of disulfide bridges from free thiols [169], this reaction depends on ROS [170] whose levels, as known, are increased in T2DM. Further studies also showed that in T2DM hyperglycemia causes membrane lipid peroxidation and osmotic fragility in red blood cells [171] leading to increase extracellular hemoglobin which directly affects the GPIbα-vWF interaction [172]. In particular, increased platelet adhesion, and microthrombi formation on fibrin(ogen), extracellular matrix, and collagen at high shear stress in the presence of free hemoglobin (≥50 mg/dL) were found. These may have implications on the shear stress-induced platelet aggregability explaining, at least in part, the increased platelet aggregation in whole blood from T2DM patients. Taking into account that T2DM patients show higher plasma concentrations of vWF, correlated with HbA1c and chronic hyperglycemia, we can suppose that the occurrence of a disturbed microhemorrheology in a diabetic environment, characterized also by elevated ROS levels, contributes to exacerbate the prothrombotic phenotype.

## 9. Role of Dyslipidemia in the Impaired Platelet Reactivity

Dyslipidemia is recognized as an independent risk factor for coronary artery and peripheral vascular disease. In this association a major role is exerted by the effects of accumulation of plasma oxidized lipids on platelet function suggesting a potential causative role for dyslipidemia in the promotion of platelet hyperreactivity in CVD [173,174]. Cholesterol accumulation in plasma membrane alters membrane structure with effects on signaling via surface receptors. Indeed, the mechanisms by which dyslipidemia promotes platelet activity and thrombosis in vivo are multiple also for the heterogeneous nature of lipoproteins.

Platelets become sensitive to a wide spectrum of interactions after low-density lipoprotein cholesterol (LDL) binding to the specific receptor on the platelet membrane: in their native form, LDLs alone do not induce platelet aggregation but increase platelet response to proaggregants; if oxidized, LDLs induce platelet aggregation also in the absence of agonist [175].

The capability of oxidized-LDL (oxLDL) particles to stimulate generation of ROS by lectin-like oxLDL receptor-1 (LOX-1) binding, a major receptor for uptake of oxLDL in endothelial cells, is one the mechanisms involved in the reduced NO bioavailability at all stages of atherosclerosis through the increases in Nox, nuclear factor kappa B (NF-κB), and mitochondrial enzymes involved in oxidative signaling [176,177]. As known, loss and/or impaired NO action can induce platelet activation, and in disease states such as hypercholesterolemia and diabetes, where ROS production is increased, a dysregulated NO metabolism becomes a critical determinant of platelet function. Indeed, diseases like hypercholesterolemia, where high levels of LDL are often accompanied by increased oxLDL, platelet hyperactivity could also depend on hyporesponsiveness to NO-related pathways [61,178,179,180]. In fact, platelets from patients with primary hypercholesterolemia, if compared with healthy controls, show higher aggregability to ADP, collagen, AA, higher ROS production, reduced sensitivity to NO, and increased activation of the proaggregant PI3K/Akt and MAPK/ERK-2 pathways. In the same individuals, platelet exposure to GLP-1, an incretin hormone with effects depending on GLP-1 influence on NO-signaling [129], does not exert any of its antiplatelet actions [178]. In this phenomenon, a role could be played by oxLDL ability to generate Nox2-derived ROS through a CD36-PKC pathway with inhibition of cGMP signaling [181], a key protective pathway activated by NO that, if overcome, results in increased platelet activation.

Platelets from patients with hypercholesterolemia show hyperaggregability, increased fibrinogen binding and surface expression of CD62P, increased production of TXA_2_ and superoxide anion, whereas plasma derived from the same patients contains increased concentrations of platelet activation markers, such as soluble sCD-40L, PF-4, sP-selectin, and β-thromboglobulin [182,183,184]. Many of these impaired platelet parameters of platelet aggregation and activation are corrected by lipid-lowering treatments.

In vitro and in vivo studies show that statins, inhibitors of 3-hydroxy-3-methylglutaryl coenzyme A (HMG-CoA) reductase and the most relevant drugs used to lower serum cholesterol levels, due to their pleiotropic effects decrease subclinical inflammation, oxidative stress, endothelial dysfunction, platelet aggregation, and activation [185,186,187,188,189], improving platelet sensitivity to NO [178], and aspirin [184], but not to GLP-1 [178]. The causes of the enhanced platelet hyperaggregability and the defective GLP-1 actions in dyslipidemia can be multifactorial, although the strong correlation with LDL underlines the role of cholesterol as a major determinant of platelet hyperreactivity with a putative role also in the impaired response to GLP-1. The modulating effects of GLP-1 on platelet function might have protective roles on the cardiovascular system, thus suggesting that a reduced and/or impaired action of GLP-1 on platelets could be involved in the platelet hyperreactivity described in metabolic disorders such as diabetes [190,191] and dyslipidemia [192,193].

Although statins represent important tools for primary and secondary prevention of CV events in hypercholesterolemia, only a low percentage of patients reach a predefined LDL target thus justifying the development of new approaches to lipid modification. At this purpose, the inhibition of proprotein convertase subtilisin/kexin type 9 (PCSK9) to reduce plasma LDL is a new approach for the treatment of hypercholesterolemia because it allows us to address the unmet clinical needs of achieving goal LDL levels for the majority of patients with high CV risk. PCSK9 is a major regulator of LDL levels as it promotes the degradation of hepatic LDL receptors, thus its inhibition causes an increase of LDL receptor activity and more circulating LDL is removed [194].

It has been recently shown that in primary hypercholesterolemia the in vivo treatment with PCSK9 inhibitors, beyond their lipid-lowering action, had important inhibitory effects on platelet aggregation and activation [129]. Given the activating direct effect of PCSK9 on platelets [195] and the relationship between PCSK9 and higher platelet reactivity [196,197,198], it is plausible that PCSK9 can directly influence platelet reactivity, thus PCSK9 inhibitors also would reduce the direct PCSK9 stimulatory effects on platelets.

The typical dyslipidemia in patients with T2DM and/or the MS is characterized by increased plasma TG concentration and low HDL concentration. In such context, the presence of small, dense LDL, more prone to oxidation, leads to a mixed atherogenic dyslipidemia.

Even though LDLs affect platelet function by modulating platelet activity more strongly than hypertriglyceridemia [173,199], there is evidence that TG-rich particles can directly activate platelets [200].

HDL has been shown to mediate various antithrombotic effects [201]. The infusions of reconstituted HDL decreased platelet activation in diabetic subjects [202]; on the contrary, in condition of impaired delivery of cholesterol by HDL from plasma and peripheral tissues, marked increases of platelet activation and thrombosis have been found [203].

## 10. Hypertension and Platelets

Arterial hypertension is one of the most important worldwide public-health challenges because of its high frequency and a leading preventable cause of premature death. Indeed, hypertension is a multifactorial disease, often clustering with other components of metabolic syndrome such as obesity, dyslipidemia, and insulin-resistance [204] and platelet activation is deeply involved in at least half of deaths due to heart disease and stroke [205,206,207].

Changes in the biochemical and functional profile of plasma membrane of platelets from hypertensive subjects are suggestive of platelet activation [208]. The increase of shear forces due to elevated blood pressure, especially adjacent to the endothelium, can promote platelet activation and degranulation [209].

Impaired NO availability, increased oxidative stress, altered Ca^++^ metabolism [210], and membrane permeability [211] are some of platelet abnormalities observed in hypertensive patients.

Vascular and endothelial dysfunction are linked to arterial hypertension and may result in a greater propensity for platelets to cause thrombosis. Actually, given the central role of endothelial cells in avoiding platelet adhesion and maintaining normal platelet function, the presence of dysfunctional endothelium would promote platelet adhesion and activation.

An important consequence of endothelial dysfunction is the reduced bioavailability of NO, a key molecule for CV health. This may be a consequence of the endothelial nitric oxide synthase (eNOS) polymorphisms [212], reduced NO production or increased breakdown of NO by ROS [213,214]. In particular, the uncoupled state of eNOS leads to a decrease in NO synthesis and increase in ROS production. The quenching of NO by superoxide anions contributes to impaired vascular smooth muscle cell response [215] and relaxation [216]. Platelets express both the constitutive eNOS, and inducible NOS (iNOS) with distinct molecular structure and characteristics [217]. The constitutive, calcium-dependent eNOS is responsible for NO production in platelets, which in turn inhibits platelet activation and aggregation by increasing cGMP levels [218]. Increase in intraplatelet Ca^++^ [219] and decrease of NO bioavailability [210] could explain, at least partially, the higher platelet aggregation observed in hypertension.

Stimulated platelets release vascular endothelial growth factor (VEGF) [220], one of the most potent angiogenic factors, and elevated VEGF levels have been found in patients with atherosclerotic risk factors, including hypertension [221,222,223]. The association between sP-selectin and VEGF levels corroborates the hypothesis that platelets are likely to be a relevant source of VEGF in hypertension; in this setting, aspirin inhibits the agonist-induced platelet aggregation and also VEGF release [224].

Intracellular Ca^++^ and Na^+^ contents can modify membrane fluidity and microviscosity that, in turn, can influence receptor functions or enzyme activities [225]. Indeed, arterial hypertension is characterized by a number of structural and functional alterations of the cell membrane including changes in membrane permeability, signal transduction, ion transport, receptor functions, because of plasma membrane differential composition, which in turn might disturb the asymmetry of the platelet plasma membrane [226,227]. Recently, structural and biochemical abnormalities in the platelet membrane from hypertensive subjects have been confirmed by studies showing an overexpression of the epithelial sodium channel [228] involved in the regulation of extracellular fluid volume and blood pressure and dispensable in platelets for migration, alpha- and dense-granule secretion and platelet collagen activation [211].

## 11. Conclusions

Platelets are key players in the thrombotic process in patients with metabolic abnormalities associated with increased risk of CVD.

This review provides an overview of changes in platelet function occurring in metabolic and hemodynamic disorders mainly characterizing the MS, all with an impact on the risk of CV morbidity and mortality owing to atherothrombotic events. Many of impairments in platelets converge on oxidative stress with release of oxidation products, which have a causal link to platelet hyperaggregability and hyperactivation. The excess of adipose tissue of the trunk and/or abdomen has a strong impact on vascular complications, through the production of PMPs and mediators with paracrine and endocrine actions, which influence platelet response. Platelet indices and biomarkers of platelet activation may have useful clinical value through the whole journey of cardiometabolic diseases for prediction and risk assessment of thrombotic risk. Different methodological approaches for platelet (dys) function investigations are now available and each-one based on different operating principles. However, few assays are able to assess “all in one device” platelet aggregation and activation pathways and standardization and quality controls are still limited despite several efforts.

Insulin resistance, a condition frequently associated with obesity, with or without hyperglycemia, dyslipidemia, and hypertension alters a number of distinct aspects of hemostasis responsible for platelets more prone to aggregate to agonists and less responsive to platelet inhibitors. However, weight reduction is a powerful measure to restore a physiological platelet function in obese subjects.

## Figures and Tables

**Table 1:** Markers of platelet activation in obesity.

Markers
Mean Platelet Volume
Thromboxane B_2_
Prostaglandin F2α
Soluble P-Selectin
Soluble CD40L
Platelet-derived Microparticles
